# Occurrence of Endoparasites in Creole Goats Under an Extensive Production System on the Southern Coast of Peru

**DOI:** 10.3390/pathogens14050437

**Published:** 2025-04-30

**Authors:** Emmanuel Sessarego, Jhony Soca-Jorge, Jose Teran, María Dávalos-Almeyda, Justo Valdivia-Zevallos, Jose Ruiz, Juancarlos Cruz, Danny Julio Cruz

**Affiliations:** 1 Estación Experimental Agraria Chincha, Instituto Nacional de Innovación Agraria, Ica 11770, Peru; e.sessarego14@gmail.com (E.S.); mvjsocaj@gmail.com (J.S.-J.); jteran@inia.gob.pe (J.T.); 2 Dirección de Supervisión y Monitoreo en las Estaciones Experimentales Agrarias, Instituto Nacional de Innovación Agraria, Lima 15024, Peru; jaruizch@gmail.com (J.R.); jcruz@inia.gob.pe (J.C.); 3 Facultad de Medicina Veterinaria y Zootecnia, Universidad Nacional San Luis Gonzaga, Ica 11702, Peru; maria.davalos@unica.edu.pe; 4 Escuela de Medicina Veterinaria y Zootecnia, Universidad Privada San Juan Bautista, Ica 11004, Peru; justo.valdivia@upsjb.edu.pe; 5 Departamento de Producción Animal, Facultad de Agronomía, Universidad de Buenos Aires, Buenos Aires C1417, Argentina

**Keywords:** risk factors, feeding type, body condition, age

## Abstract

Endoparasitosis is a critical health challenge in the management of Creole goats under extensive production systems due to its negative impact on animal health and productivity. This study determined the occurrence of endoparasites and identified associated risk factors in Creole goats from the southern coast of Peru. Fecal samples were collected from 129 goats in two localities of the Pisco province to detect the presence of oocysts from *Eimeria* spp. and eggs from trichostrongyles, *Skrjabinema* spp., *Trichuris* spp., and *Fasciola hepatica*. Data were analyzed using logistic regression models and adjusted through bootstrapping and stepwise selection methods, with locality, feeding type, age, and body condition as predictive variables. The results revealed a high occurrence of *Eimeria* spp. (86.0%) and trichostrongyles (65.1%), while *Fasciola hepatica* (14.0%) and *Skrjabinema* spp. (7.0%) were exclusively identified in Independencia and San Clemente, respectively. Mixed infestations were predominant (65.9%), occurring more frequently in Independencia (75.9%) than in San Clemente (57.7%) (OR: 2.26, *p* < 0.05). The likelihood of infestation was significantly higher in Independencia for *Eimeria* spp. (OR: 5.72, *p* < 0.01) and *Fasciola hepatica* (OR: 61.4, *p* < 0.01). Moreover, goats fed exclusively on alfalfa were more likely to be infested with *Fasciola hepatica* compared to those fed a mixed diet of alfalfa and crop residues (OR: 0.06, *p* < 0.05). These findings underscore the necessity of implementing comprehensive health programs tailored to local management and feeding conditions.

## 1. Introduction

Goats were among the first species domesticated by humans and are known for their adaptability to diverse geographic regions, with higher concentrations in tropical and arid areas [1]. In Peru, goats thrive in coastal regions due to the favorable climatic conditions. As of 2021, the national goat population was estimated at approximately 1,801,257 animals, with 4.17% located in the Ica region [2]. This population exhibits broad phenotypic variability, including composite coat colors, beards, horns, and parallel, normally developed teats. Dual-purpose biotypes have been described, with a milk production capacity of approximately 1 to 2 L per day and an average adult weight of 45.30 ± 0.99 kg, highlighting their adaptation to local conditions and their role in extensive production systems [3,4].

Although studies have characterized the phenotypic and productive traits of Creole goats, research on their health and genetics has been more limited. In recent years, interest in understanding their disease resistance and genetic conservation value has grown. This has led to state-funded research focused on their characterization and preservation, aiming to improve their management and conservation in production systems.

Goats play a vital role in rural economies, particularly in arid areas, but face numerous challenges that limit their productivity [5,6]. One major challenge in goat farming is health problems caused by endoparasites such as protozoans, nematodes, and trematodes [7,8]. Parasitic infestations lead to growth retardation, weight loss, reduced milk production, and even abortions in severe cases, significantly impacting animal health [9,10]. Among these parasites, *Fasciola hepatica*, a trematode of zoonotic importance, poses an additional risk due to its implications for public health [11].

The extent of parasitic infestation impacts depends largely on the management system [12]. Poor practices, such as the absence of health programs or deworming schedules, exacerbate the problem [13,14]. This issue is particularly critical in extensive farming systems, where management practices are less controlled and health resources are often limited [15,16]. Furthermore, a lack of knowledge regarding the occurrence of endoparasitic diseases complicates the development of effective control strategies. Consequently, deworming is often carried out without considering appropriate dosages based on parasite load or the animal’s live weight, or it is carried out using unsuitable active ingredients, which promotes the development of antiparasitic resistance over time [17,18]. Despite this, there is no updated information, and studies quantifying the economic losses caused by these infections in goat farming in Peru are still lacking.

Given the scarcity of studies on endoparasites in Creole goats from the southern coast of Peru, this research aimed to determine their occurrence and identify associated risk factors, including locality, feeding type, age, and body condition, thus contributing to the development of more effective control and health management strategies.

## 2. Materials and Methods

### 2.1. Study Area

**Figure 1:**
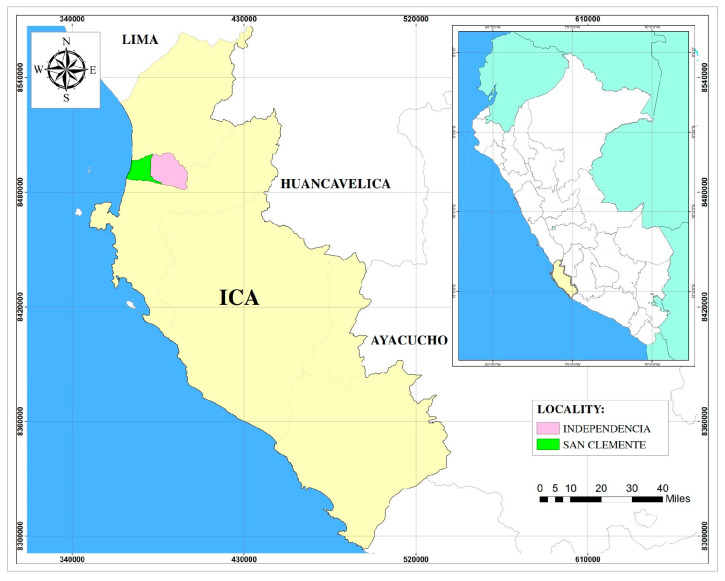
Location of the Independencia and San Clemente within the Ica region, Peru.

This study was conducted during the winter season (May–June) in the localities of Independencia (latitude: 13°38′49″ S; longitude: 76°0′47″ W; altitude: 211 m) and San Clemente (latitude: 13°38′32″ S; longitude: 76°8′35″ W; altitude: 76 m), both located in the province of Pisco, Ica region, in southern Peru (Figure 1). These areas are home to the largest goat population in the region [2] and are characterized by an arid subtropical climate, with temperatures ranging from 16 to 24 °C and a relative humidity of 85% during winter and from 19 to 27 °C with 72% humidity in the months preceding this period.

### 2.2. System of Production and Management

Goat rearing in the Ica region is primarily concentrated in Independencia and San Clemente. It is characterized by an extensive system with seasonal reproductive periods, which includes a breeding campaign from October to December and kidding from March to May. Goats are raised for milk production, with a retention limit of up to the fourth kidding or, in extreme cases, up to the fifth. Some producers practice transhumance, which allows the animals to consume natural pastures and native shrubs, such as huarango (*Prosopis pallida*), while most rely on alfalfa-based diets, with or without crop residues (corn, cotton, or cassava); this practice is more pronounced in San Clemente than in Independencia. Deworming practices are carried out annually, with antiparasitic drugs containing the active ingredients triclabendazole, albendazole, and mebendazole being applied during the postpartum period.

### 2.3. Study Population

Samples were collected from 129 Creole goats selected from herds with similar management practices and accessible producer participation. The sampling size was determined by non-probability sampling for presence detection.

The sampled goats had between one and four kiddings and were in their first third of lactation. The average weight was 41.00 ± 2.1 kg. Of these, 71 belonged to six herds in San Clemente and 58 to five herds in Independencia. These herds were representative and managed without association with other livestock species. Herds were selected based on an alfalfa-based feeding regimen or a combination of alfalfa and crop residues. Sampling took place in May, the month with the highest reported incidence of parasitosis according to producer observations. No deworming was performed prior to sample collection.

Body condition was assessed using a 1 to 5 scale through palpation and visual inspection, considering muscle mass, fat deposits, and connective tissue at the base of the tail, trunk, and prominent bony structures. The age of the goats was determined through dental chronology, ranging from four permanent incisors to full dentition.

### 2.4. Sample Collection

A total of 129 fecal samples were collected directly from the rectum of the goats, with each sample weighing approximately 10 g. The samples were stored in labeled containers and kept in a cooler at a temperature of 4–8 °C [19]. They were then transported to the parasitology laboratories of Escuela Profesional de Medicina Veterinaria y Zootecnia at the Universidad Privada San Juan Bautista and Facultad de Medicina Veterinaria y Zootecnia at the Universidad Nacional San Luis Gonzaga, both located in the Ica region, southern Peru.

### 2.5. Coprological Examination

The techniques employed to determine the occurrence of gastrointestinal parasites were qualitative methods with high sensitivity and specificity. Nematode and cestode eggs and protozoan oocysts were detected using Sheather’s flotation technique [20]. Briefly, 2 g of feces was mixed with 10 mL of Sheather’s solution, thoroughly homogenized, and filtered into a test tube. The mixture was then centrifuged at 1500–2000 rpm, and a sample of the supernatant was placed on a slide, covered with a cover slip, and examined under a microscope to identify the eggs and oocysts.

Egg identification was based on shape, size, coloration, protective membrane, presence of an operculum, and internal segmentation [21]. Nematode eggs, such as trichostrongyles and *Skrjabinema* spp., are oval, transparent, and with a thin protective membrane, with *Skrjabinema* spp. being smaller. *Trichuris* spp. eggs are elliptical, yellowish-brown, with well-defined polar plugs, while oocysts of *Eimeria* spp. are small, oval or spherical, and with a smooth protective membrane and no internal segmentation, characteristic of protozoans. No cestode eggs were found in the samples.

For *Fasciola hepatica* eggs, the modified Dennis sedimentation technique was employed [22]. An amount of 5 grams of feces was mixed with 20 mL of a detergent solution (5 g of detergent per liter of water) in a sedimentation tube, filtered through gauze to remove large particles, and allowed to settle for 30 min. Half of the supernatant was decanted, and fresh detergent solution was added. This washing process was repeated three times until only sediment remained, and a sample of the sediment was then examined under a microscope.

Protozoan oocysts and eggs of nematodes, cestodes, and trematodes were observed and identified using 10× and 40× objectives.

### 2.6. Statistical Analysis

Occurrence was calculated using the formula established by Rafiq et al. [23]:

$$O(\%)=\frac{n+}{n}\times 100$$

where **O** (%) represents occurrence as a percentage, **n+** is the number of animals that tested positive for the evaluated parasite genus (based on the presence of eggs in microscopic observation), and **n** corresponds to the total number of animals evaluated in each study group. Occurrence rates are reported with 95% confidence intervals (95% CI) to reflect the uncertainty of the estimates.

The presence or absence of endoparasites of the genera *Eimeria* spp., trichostrongyles, *Skrjabinema* spp., *Trichuris* spp., and *Fasciola hepatica* was analyzed using a logistic regression model. The risk factors considered were the type of feed (forage or forage + crop residues), locality (San Clemente or Independencia), body condition (included as a continuous covariate), and age (less than or greater than 4 years).

Given the limited sample size, the most appropriate model adjustment was performed using bootstrapping methodology, with 2000 iterations implemented through the caret library [24]. This procedure yielded robust estimates by adequately capturing sample variability, and preliminary tests confirmed the stability of the parameters. Variable selection was performed using a stepwise procedure based on the Akaike Information Criterion (AIC), which adds or removes variables to reduce the AIC, balancing model complexity with goodness of fit and avoiding overfitting. The selected risk factors were incorporated into the final model, which was applied to the original dataset to find the association between these factors and infestation occurrence.

To evaluate the performance of each model, a confusion matrix was generated using a classification threshold of 0.5, yielding an accuracy greater than 0.65, which indicates that the model adequately distinguishes between cases with and without infestation.

Finally, for each predictor variable in the final model, odds ratios (ORs) were calculated by exponentiating the coefficients (β). Contrasts between factor levels were performed using the emmeans library [25], which enabled evaluation of predicted probability differences and calculation of adjusted marginal means. All statistical analyses were conducted using R software version 4.4.2 [26].

## 3. Results

### 3.1. Occurrence of Endoparasites

The occurrence of the evaluated parasitic genera showed marked variability, with *Eimeria* spp. (86.0%) and trichostrongyles (65.1%) being the most frequent in Creole goats, as shown in Table 1. At the locality level, trichostrongyles was more prevalent in San Clemente (67.6%) compared to Independencia (62.1%). *Fasciola hepatica* was detected exclusively in Independencia, with an occurrence of 14.0%, while *Skrjabinema* spp. was identified only in San Clemente, with a low occurrence of 7.0%.

Mixed infestations were the most frequent, accounting for 65.9% of total cases, while single infestations represented 30.2%. This indicates that mixed infestations were 2.18 times more frequent than single infestations, with an occurrence 3.38 times higher in Independencia and 1.58 times higher in San Clemente. The most common combination was *Eimeria* spp. + trichostrongyles, representing 65.9% of mixed infestations, whereas other combinations, such as *Eimeria* spp. + *Trichuris* spp. and trichostrongyles + *Fasciola hepatica*, showed lower occurrence.

### 3.2. Risk Factors for Endoparasite Presence

Statistical models revealed that locality, body condition, type of feed, and age were key factors influencing the likelihood of infestation by different parasitic genera, as shown in Table 2. The locality of Independencia exhibited a significantly higher probability of infestation by *Eimeria* spp. (95.7%; 95% CI: 86.8–98.7%) compared to San Clemente (79.4%; 95% CI: 68.0–87.4%), with an OR 5.72 times greater (*p* < 0.05). For *Fasciola hepatica*, the odds were significantly higher in Independencia (*p* < 0.05), with an infestation probability of 13.9% (95% CI: 3.7–40.4%), compared to 0.26% (95% CI: 0.01–5.4%) in San Clemente.

Animals fed exclusively on alfalfa were at greater risk of *Fasciola hepatica* infestation. Body condition significantly influenced the likelihood of infestation by *Eimeria* spp., with animals in poorer condition being more likely to be infested (*p* < 0.05). Age also had a significant effect on infestation by *Trichuris* spp.; animals aged four years or younger had an infestation probability of 1.3% (95% CI: 0.26–6.3%) compared to 15.6% (95% CI: 4.5–42.0%) in older animals, with an OR 0.071 times lower (*p* < 0.05).

Mixed infestations were more common in Independencia (75.4%; 95% CI: 62.8–84.8%) than in San Clemente (57.6%; 95% CI: 45.9–68.6%), with an OR of 2.26 (*p* < 0.05).

## 4. Discussion

The results are largely influenced by environmental and management factors. The onset of the rainy season increases humidity (72% to 85%) and provides an optimal temperature range (16–24 °C), which generates favorable conditions for endoparasite development. During this period, goats tend to cluster together more due to the lower thermal sensation, facilitating the transmission of parasites by close contact and fecal contamination [27]. In addition, the sampled goats were within a month of the start of the kidding season, a phase associated with physiological stress that increases their susceptibility to parasitic infections [28]. These combined factors could contribute to an increased risk of parasitic infestation in the herds.

The high occurrence of *Eimeria* spp. and trichostrongyles in Creole goats aligns with studies conducted in tropical and arid climates, where these genera dominate due to their adaptability to different environmental conditions [29,30,31]. While these results offer relevant insights, they should be considered in light of the sample size. The exclusive detection of *Fasciola hepatica* in Independencia is associated with the presence of wetlands in this area, which support the development of the intermediate host for this parasite [32]. In contrast, the identification of *Skrjabinema* spp. only in San Clemente, albeit with low occurrence, may reflect local differences in grazing management.

The predominance of mixed infestations, particularly the combination of *Eimeria* spp. + trichostrongyles, highlights the coexistence of different parasites within a single host, which negatively impacts animal health [33,34]. This pattern is consistent with findings in similar extensive systems, where prolonged exposure to contaminated environments facilitates the persistence of predominant genera [35,36].

The higher probability of infestation by *Eimeria* spp. in Independencia can be attributed to specific characteristics of this locality, such as higher animal density, and the presence of wetlands [37,38]. Similarly, the higher occurrence of *Fasciola hepatica* in Independencia is linked to the presence of wetlands, which provide ideal habitats for snails of the genus *Lymnaea*, intermediate hosts of *Fasciola hepatica* [32,39]. The association between alfalfa feeding and *Fasciola hepatica* infestation is consistent with studies indicating that irrigation practices in alfalfa cultivation create favorable conditions for the parasite’s infective stages [40,41]. In contrast, the use of crop residues, which are handled and sun-dried post-harvest, alters temperature conditions, disrupting the parasite’s life cycle while also improving the host’s nutritional balance [42].

The influence of body condition on *Eimeria* spp. infestation suggests that malnourished animals lack the metabolic resources necessary for an effective immune response, making them more susceptible to parasitic infections [43]. The age-related increase in *Trichuris* spp. infestation may reflect cumulative exposure in older animals, increasing their risk of persistent subclinical infections [44,45].

The higher occurrence of mixed infestations in Independencia compared to San Clemente may be due to high humidity, overcrowding, and the presence of wetlands, which promote the accumulation of infective stages in the environment [34]. These mixed infestations have detrimental effects on animal health, as interactions between parasites compromise the nutritional and immune status of the host [33].

Endoparasitosis should not be considered a minor issue. According to Sarkar et al. [46], it causes hematological changes, such as a decrease in red blood cell count and hemoglobin levels, indicative of anemia, as well as biochemical alterations, including a reduction in total protein and albumin levels, which are associated with malnutrition or malabsorption. These effects demonstrate the negative impact of endoparasites on the intestinal, nutritional, and immune health of goats.

Information on the productive and economic aspects related to parasitic infections in Peruvian creole goats is limited, as they have been little studied. However, parasitic infections can affect production and reproductive efficiency, as has been reported in other environments [47,48]. This reduces the overall performance of the herd and impacts the producers’ economy, through both decreased production and the additional costs associated with anthelmintic treatments [49].

This study has some limitations. The small sample size may affect the generalization of results, although statistical methods were applied to reduce this impact. Additionally, the lack of species differentiation within *Eimeria* and trichostrongyles limits conclusions about their pathogenic impact. To improve parasitological research in these production systems, future studies should include molecular or morphological identification in a larger population, allowing for a better understanding of endoparasite diversity and clinical effects.

## 5. Conclusions

The high occurrence of *Eimeria* spp. and trichostrongyles observed in this study, along with the influence of various risk factors on the presence of *Eimeria* spp. and *Fasciola hepatica*, demonstrates the fundamental role of environmental and management conditions in Independencia in the dynamics of endoparasitosis compared to San Clemente. This highlights the need to implement prevention and control strategies adapted to local conditions to mitigate the impact of these parasites on animal health. These strategies have to include the strategic rotation of grazing and resting areas to reduce the load of infectious stages; the rational use of anthelmintics based on prior coproparasitological studies, with applications two to three times per year depending on fecal analysis results; and improvements in water management and dietary diversification.

## Figures and Tables

**Table 1:** Occurrence of endoparasites in Creole goats by locality, parasite genus, and type of infestation (total number of animals evaluated, n = 129).

Parasites	Occurrence: % (n+/n; 95% CI)		
	Independencia (n = 58)	San Clemente (n = 71)	Total (n = 129)
By type			
*Eimeria* spp.	94.8 (55/58; 85.9–98.2)	78.9 (56/71; 85.9–98.2)	86.0 (110/129; 79.0–91.0)
Trichostrongyles	62.1 (36/58; 49.2–73.4)	67.6 (48/71; 56.1–77.3)	65.1 (84/129; 56.6–72.8)
*Skrjabinema* spp.	0.0 (0/58; 0.0–6.2)	12.7 (9/71; 6.8–22.4)	7.0 (9/129; 3.7–12.7)
*Trichuris* spp.	1.7 (1/58; 0.3–9.7)	2.8 (2/71; 0.7–9.7)	2.3 (3/129; 0.7–6.6)
*Fasciola hepatica*	31.0 (18/58; 20.6–43.8)	0.0 (0/71; 0.0–5.1)	14.0 (18/129; 9.0–21.0)
By infestation type			
Simple	22.4 (13/58; 13.6–34.7)	36.6 (26/71; 26.4–48.2)	30.2 (39/129; 23.0–38.6)
*Eimeria* spp.	20.7(12/58; 12.3–32.8)	21.1(15/71; 13.2–40.0)	20.9(27/129; 14.8–28.7)
Trichostrongyles	0.0 (0/58; 0.0–6.2)	15.5(11/71; 8.9–25.7)	8.5 (11/129; 4.8–14.6)
*Fasciola hepatica*	1.7 (1/58; 0.3–9.1)	0.0 (0/71; 0.0–5.1)	0.7 (1/129; 0.1–4.2)
Mixed	75.9 (44/58; 63.5–85.0)	57.7 (41/71; 46.2–68.5)	65.9 (85/129; 57.4–73.5)
*Eimeria* spp. + trichostrongyles	44.8 (26/58; 32.7–57.5)	42.3 (30/71; 31.5–53.8)	43.4 (56/129; 35.2–52.0)
*Eimeria* spp. + *Skrjabinema* spp.	0.0 (0/58; 0.0–6.2)	4.2 (3/71; 1.4–11.7)	2.3 (3/129; 0.8–6.6)
*Eimeria* spp. + *Trichuris* spp.	0.0 (0/58; 0.0–6.2)	1.4 (1/71; 0.2–7.6)	0.7 (1/129; 0.1–4.2)
*Eimeria* spp. + *Fasciola hepatica.*	13.8 (8/58; 7.2–24.9)	0.0 (0/71; 0.0–5.1)	6.2 (8/129; 3.2–11.8)
Trichostrongyles + *Fasciola hepatica*	1.7 (1/58; 0.3–9.1)	0.0 (0/71; 0.0–5.1)	0.7 (1/129; 0.1–4.2)
*Eimeria* spp. + trichostrongyles + *Fasciola hepatica*	13.8 (8/58; 7.2–24.9)	0.0 (0/71; 0.0–5.1)	6.2 (8/129; 3.2–11.8)
*Eimeria* spp. + trichostrongyles + *Skrjabinema* spp.	0.0 (0/58; 0.0–6.2)	8.4 (6/71; 3.9–17.2)	4.7 (6/129; 2.1–9.8)
*Eimeria* spp. + trichostrongyles + *Trichuris* spp.	1.7 (1/58; 0.3–9.1)	1.4 (1/71; 0.2–7.6)	1.6 (2/129; 0.4–5.5)

**n+**: positive cases; **n**: number of animals in each study group.

**Table 2:** Odds ratios (OR) from logistic regression evaluating the risk factors associated with the presence of endoparasite genera in Creole goats.

Factors	*Eimeria* spp.	Trichostrongyles	*Skrjabinema* spp.	*Trichuris* spp.	*Fasciola hepatica*	Mixed
Location						
Independencia/San Clemente	5.72 **	-	0.06	-	61.4 **	2.26 *
Type of feed						
Alfalfa/alfalfa + stubble	-	1.89	-	-	0.06 *	-
Age						
≤4 years/>4 years	-	-	-	0.07 *	-	-
Body condition	0.084 *	-	-	-	-	-
AIC	98.17	168.63	56.19	27.24	67.57	164.82
BIC	106.74	174.35	70.49	32.96	76.16	170.54

* and ** indicate statistical significance at 95% (*p* < 0.05) and 99% (*p* < 0.01), respectively. AIC: Akaike Information Criterion; BIC: Bayesian Information Criterion.

## Data Availability

The data presented in this study are available upon request from the corresponding author. The data belong to INIA under the PROCAP project and are only accessible upon request to the authors due to project-specific restrictions.
